# Procedural Moral Enhancement

**DOI:** 10.1007/s12152-016-9258-7

**Published:** 2016-04-20

**Authors:** G. Owen Schaefer, Julian Savulescu

**Affiliations:** 1 Centre for Biomedical Ethics, Yong Loo Lin School of Medicine, National University of Singapore, Block MD11, Clinical Research Centre, 10 Medical Drive, Singapore, 117597 Singapore; 2 Uehiro Centre for Practical Ethics, Faculty of Philosophy, Oxford University, Suite 8, Littlegate House, 16/17 St Ebbe’s St, Oxford, OX1 1PT UK

**Keywords:** Moral enhancement, Reliability, Rawls, Proceduralism

## Abstract

While philosophers are often concerned with the conditions for moral knowledge or justification, in practice something arguably less demanding is just as, if not more, important – reliably making correct moral judgments. Judges and juries should hand down fair sentences, government officials should decide on just laws, members of ethics committees should make sound recommendations, and so on. We want such agents, more often than not and as often as possible, to make the right decisions. The purpose of this paper is to propose a method of enhancing the moral reliability of such agents. In particular, we advocate for a procedural approach; certain internal processes generally contribute to people’s moral reliability. Building on the early work of Rawls, we identify several particular factors related to moral reasoning that are specific enough to be the target of practical intervention: logical competence, conceptual understanding, empirical competence, openness, empathy and bias. Improving on these processes can in turn make people more morally reliable in a variety of contexts and has implications for recent debates over moral enhancement.

## Introduction

While moral and political philosophers are often concerned with high-level issues concerning the conditions of goodness and justice, in practice individual judgment plays a significant role in realizing the demands of morality. Individual judges and juries hand down purportedly fair sentences, government officials decide on just laws, and members of the public make personal decisions on whom to elect. In order to realize just outcomes, we need such agents, more often than not and as often as possible, to make the right decisions. While there is no doubt that interaction between agents in groups is important to deliberative quality (see, e.g., [1]), the purpose of this paper is to propose a method of enhancing the moral decision-making of individual agents. In particular, we advocate for a procedural approach; certain internal processes generally contribute to people’s moral reliability. Building on the early work of Rawls, we identify several particular factors related to moral reasoning that are specific enough to be the target of practical intervention: logical competence, conceptual understanding, empirical competence, openness, empathy and bias. Improving on these processes can in turn make people more morally reliable.^1^

To clarify: By ‘reliability’, we just mean the likelihood of agents to come to the right moral conclusions, rather than the stronger sort of reliability necessary for some conceptions of knowledge and/or justification (see, e.g., [3–5]). This is not meant to beg the question in favor of moral realism; reliability can be cashed out in terms of quasi-realist conceptions of ‘good’ and ‘right’ (where the terms do not refer to objective, mind-independent facts). Complications will emerge concerning how to assess reliability procedurally, where the correctness of certain outputs are not presupposed. These are addressed in the ‘garbage in, garbage out’ section below.

Procedural analysis is not the only possible approach to moral reliability, but it has the advantage of avoiding commitment to a wide set of substantive moral claims. Some of those claims would inevitably be controversial. The account we propose below will, we believe, be generally acceptable across a wide array of normative and meta-ethical theories. Some substantive commitments (e.g., concerning clearly irrelevant moral considerations) may have to be made along the way, but those commitments are rather minimal. By avoiding most of these presuppositions, we can sidestep some objections to moral enhancement based on the issue of imposing moral values, e.g., from Harris [6] and Jotterand [7]. Indeed, Harris in passing explicitly accepts the relevance of some of the sorts of capacities we emphasize (e.g., empirical and logical competence) for moral reliability. And for a longer argument for how a reasoning-based approach may be amenable to the virtue ethics Jotterand supports, see Fröding [8].

Moreover, a procedural approach avoids begging the question for or against particular views of morality. A more substantive approach where reliable agents were identified based on how frequently they produced the right moral judgments would require us to prejudge the content of those moral judgments. But this will often mean presupposing an answer to questions of morality that particular agents were tasked with determining. And it also opens up a regress problem – why should we think that the individual assessing reliability of the agents’ judgments are themselves reliable?^2^

Nevertheless, procedural approaches in general, and the particular approach we develop, are not incompatible with other accounts of reliability. We do not aim at setting up necessary and sufficient conditions for moral reliability. Rather, we will identify several procedural capacities that can contribute to greater reliability. This does not exclude other, additional factors that can contribute to reliability. In fact, someone who fulfills all the criteria we lay out may still be – all things considered – morally unreliable, and conversely someone who is incompetent at all of them may be reliable. This analysis is still useful, though, to the extent that improvement in the capacities we lay out will generally lead to greater reliability – they will be more likely to make correct moral judgments than if they lacked those capacities.

A further desideratum in our account is applicability. We would like to identify factors of moral reliability that can potentially be deployed, both in the selection and improvement of agents. Procedures that are too abstract or high-level will not be terribly useful in practical scenarios such as the selection of ethics committee members. At the same time, our purpose is not to recommend certain particular tests of the capacities we identify. Whether a given test or intervention is effective in one of the domain we identify is an empirical matter. What we do seek to offer are clear standards for evaluating such interventions, and suggest a direction for future research into developing novel methods of determining and improving agents’ moral reliability.

This essay will be structured as follows. Rawlsian Competent Judges section explains why Rawls’ early work contains a good starting point, fulfilling the main desiderata of an account of procedural moral enhancement. A Modified Rawlsian Approach section offers several important modifications of Rawls’ account, expanding it and showing how it can be made suitable as a general account of procedural moral enhancement – though one that does not require commitment to Rawls’ broader moral framework. An objection (‘garbage in, garbage out’) to our approach is considered and addressed in Garbage In, Garbage Out? section. The final Conclusions section is a brief conclusion to the arguments put forward in this paper.

## Rawlsian Competent Judges

What we are looking for is an account of moral enhancement that 1) is procedural (thus avoiding many question-begging moral assumptions); 2) outlines conditions under which people’s moral judgments are more reliable; and 3) is detailed enough to offer practical guidance. An account that meets all these criteria can be found in the early Rawls; we will briefly outline it here, and explain how it is suitable at least as a starting point for an account of procedural moral reliability.

The account comes from [9] paper, “Outline of a Decision Procedure for Ethics”. This was one of Rawls’ earliest works, one that in some ways lays the groundwork for the method of reflective equilibrium developed more fully in *A Theory of Justice* [10]. The early paper’s aim is explicitly one of developing a decision procedure; even if it is a good one, such a procedure would be of little help in identifying morally reliable agents. However, an early step in developing that decision procedure is identifying “competent judges.” The overall chain goes roughly as follows: 1) Identify the class of competent judges; 2) identify the class of considered judgments, which are a subset of the judgments of competent judges (subject to a series of constraints); 3) formulate a principled explication of all such considered judgments; 4) ensure such principles are reasonable/justifiable. The resulting principles are taken to be morally sound. Throughout, judgments themselves are doing most of the work – principles are built up from the judgements themselves, and competent judges only come into the story insofar as they generate considered judgments (and, perhaps, agreement among them lends credence to a proposed principle). Still, the idea of competent judges can be isolated from the remaining structure and instead repurposed in order to develop an account of moral reliability.

We can go through each of the three desirable criteria of an account of moral enhancement (procedural, reliable and practical) in turn and see how Rawls’ account of competent judges meets each. First and foremost, his account is procedural. Rawls is quite explicit on this point: “Competence is determined solely by the possession of certain characteristics...[A] competent judge must not be defined in terms of what he says or by what principles he uses.” ([9], p. 180) Rawls’ reason for proceduralism is similar to our own; he does not want his account to presuppose certain moral principles. The purpose of his account, after all, is to figure out a way to determine moral principles. While there is not the same sort of strict circularity problem in developing an account of moral reliability, it will still be similarly desirable to avoid a set of substantive, controversial assumptions.

Second, while Rawls envisions a wider overall decision-procedure to generate good judgments, the judgments of competent agents are taken to be reliable on their own. Rawls is interested in agents who are especially competent at “coming to know” certain moral truths. In this way, “competent judges are those persons most likely to make correct decisions.” ([9], p. 183) The further criteria of particular judgment and refinement of principles might serve to improve upon that competence – considered judgments are a further subclass of opinions that are especially likely to be correct. Even so, the broad class of all judgments (both considered and non-considered) of competent judges would be reliable on this account. Or at least, they would be more reliable than non-competent judges. And that relative claim is all we need for present purposes. When trying to decide which of various individuals should be given responsibility over an ethical matter, relative moral competence is a perfectly acceptable criterion.

Third, with proper refinement, Rawls’ account is suitably detailed to serve as a practical standard for moral reliability. To a certain extent, Rawls hedges on just how practical his account of competent judges is. He admits that, in some instances, we would not be able to use his criteria to determine who, among a group, are competent judges and who are not. There will be a certain amount of vagueness. At the same time, Rawls emphasizes that there will be some individuals who excel so much on the given criteria that we cannot but recognize them as a competent judge. This is somewhat unsatisfactory, as Rawls does not go into detail concerning how, exactly, we would be able to measure (say) someone’s empathetic understanding. Part of the purpose of the next section of this paper will be to flesh out a more attractive understanding of the criteria, and in doing so suggest how these could be practically deployed.

## A Modified Rawlsian Approach

We have seen how Rawls’ account fits our criteria for a procedural account of moral reliability. But more than that is needed; the account should be convincing, if it is to be deployed in practice. Indeed, some aspects of Rawls’ account will need modification to be fully suitable, but the overall structure provides useful guidance in how to proceed.

### Intelligence

The first feature of the competent judge given by Rawls is intelligence, “which may be thought of as that ability which intelligence tests are designed to measure.” ([9], p. 178) Unfortunately, there is very little further discussion of what is encapsulated by intelligence and, importantly, why we should accept it as important to moral reliability at all. On its face, the use of intelligence tests per se as an indicator of moral reliability is unattractive. For example, quantitative competence is one central area of intelligence tests. But the ability to perform mathematical operations does not in itself clearly relate to our ability to engage in moral reasoning in most contexts.^3^ A good account of procedural reliability must link together the feature in question and the process of judgment formation.

That having been said, there is one particular feature sometimes measured by intelligence tests that is morally useful: **logical competence**. This includes the ability to make proper logical inferences and deductions, spot contradictions in one’s own beliefs and those of others, as well as formulate arguments in a way that can highlight the true point of contention between interlocutors. This is not to say that all moral reasoning must always be framed in strict premise-conclusion form. But it will be important that when reasoning *does* take an at least implicitly logical form that the form is correct – the conclusion does really follow from the premises.

Moreover, logical competence is central to a procedural account of reliability because the correct moral judgments, whatever they turn out to be, should be mutually coherent.^4^ Logical competence can help people identify the logical implications of their views. People may not realize their views are, taken together, jointly incoherent. One might hold, for instance, the following three views: all corrupt politicians should be punished no matter how mild the corruption; one’s favourite politician is mildly corrupt; and one’s favourite politician should not be punished for so mild a corruption, given all the good work she is doing. These are jointly inconsistent, as the first two views imply by *modus ponens* that one’s favourite politician should be punished even for mild corruption. Something has to give – logically, one of the views must be given up. Better understanding, implicit or explicit, of logical rules like modus ponens can help avoid these inconsistencies and force corrections.^5^

If reasoners are to properly think through the implications and contradictions of their commitments, though, it will be important to possess more than just awareness of logical rules. One should be able to, on reflection, understand and appreciate the ideas, principles, intuitions and other thoughts that are at play. Insofar as these are moral ideas, the relevant understanding will be, in part abstract – clearer **conceptual understanding** will be an asset to moral reasoners. This includes a clear understanding of moral ideas’ content, strength and scope and the ability to communicate that understanding effectively. Introspection gives one a leg up in adequately discerning the content of an idea, but people could become confused or even self-deceived which will significantly interfere with the reliability of those judgments. Strength will be crucial in helping determine which of two competing ideas to abandon, or whether a moral consideration outweighs a non-moral one. And identifying the scope of an idea – what it applies to – is necessary to ensure it is correctly deployed.

Furthermore, in order to understand the implications of a particular moral idea (say, killing is wrong), it helps to have a clear grasp of the notions involved (in this case, not just wrongness but what exactly killing constitutes). Vague and distorted ideas will lead to unreliable inferences, inducing behaviours that are not in line with someone’s considered judgments. By contrast, proper understanding of an idea will clarify and make salient the proper inferences to make. In this way, conceptual understanding aids in logical competence – though they are distinct enough to merit separate categorization. In particular, measurements of logical competence will not serve as measurements of conceptual understanding, and vice-versa.

It might seem that evaluating conceptual competence would naturally presuppose certain substantive commitments that go against the procedurals framework we are advocating. Determining what the concept of justice amounts to, for instance, will greatly shape one’s substantive views about just decisions, institutions, governments, etc. The competence identified here, though, is not to be evaluated in a question-begging way that presupposes the content, scope and strength of some normative concept. Rather, it will involve more general capacities of reflection, attention to detail, clarification and comprehension of abstract content. These capacities are meant to help individuals decide for themselves the content, strength and scope of various concepts. They will be more reliable, however, insofar as clarity of thought will help avoid errors of misunderstanding that come when a concept is vague and ill-formed.

### Empirical Competence

We can more straightforwardly adopt the second feature of Rawls’ competent judges, concerning worldly knowledge. This encompasses knowledge of “those things concerning the world about him and those consequences of frequently per-formed actions, which it is reasonable to expect the average intelligent man to know. Further, a competent judge is expected to know, in all cases whereupon he is called to express his opinion, the peculiar facts of those cases.” (ibid, p. 178) The knowledge concerns non-moral, empirical facts about the world, so we will refer to it as **empirical competence**.

Again putting aside the ‘average’ baseline, it is fairly clear how awareness of the non-moral facts on the ground can improve moral reliability. Consider the following valid moral argument:

- P1: Senator Barney accepts bribes
- P2: Anyone accepting bribes should be punished
- C: Senator Barney should be punished

P2 and the conclusion are moral claims, and so without further elaboration are untouched by empirical concerns. However, P1 is an empirical, non-moral claim. The moral conclusion only follows if it is correct. Anyone endorsing the conclusion that Senator Barney should be punished on the basis of the above reasoning needs to have good grounds for the claim that Senator Barney accepts bribes. Some sort of evidence such as a witness of the bribery will be needed. And those evaluating such evidence will need to assess a number of factors. Is the witness reliable? How do we know what was witnessed was really a bribe? What did the briber procure? Those who are generally more competent at evaluating empirical claims will more reliably ascertain the truth of P1, and in turn make more reliable evaluations of the moral question of whether Senator Barney should be punished.

This point can be generalized. Non-basic moral judgments will often rest on arguments (or something approximating arguments) with empirical premises. Empirical competence can improve people’s ability to effectively evaluate those premises, and in that way improve the reliability of the moral conclusions that rely in part on such premises. This will make improvements in people’s empirical competence an important feature of moral reliability. Arguably, this is not a strictly procedural feature of reliability; it refers to the content of particular judgments, rather than the processes involved. But the main reason for adopting a procedural approach was avoidance of controversial and question-begging moral claims in evaluating moral reliability; integrating non-moral competencies into the account also allows us to avoid such substantive moral issues.

Empirical competence is a more vague notion than logical competence, so some explication of what it involves will be useful. Like reasoning itself, empirical competence is an umbrella concept encompassing a number of different sub-capacities, and we will delineate two (this discussion is meant to be indicative of the nature of empirical competence, not exhaustive of all ways it might be improved). One aspect is long-term memory. Properly remembering prior personal observations will assist in judgments concerning personally-experienced events. For instance, if one personally witnessed Senator Barney taking what may be a bribe, accurate recollection of what actually occurred will be crucial in evaluating his culpability. Relatedly, remembering related facts such as whether Senator Barney gave the briber any favours or the content of others’ witness statement will also aid in evaluating whether a bribe actually took place. And improving memory is relatively straightforward – it is easily testable, and already has a significant body of research supporting various means of improvement.

Another relevant capacity is knowledge of an array of facts potentially relevant to moral judgment. These might be general like laws of physics or specific like the occurrence of various historical events. The range of knowledge should be wide so it can be deployed in diverse and unexpected circumstances. In the case of Senator Barney, it may involve knowledge of what constitutes bribery. This is closely related to conceptual understanding discussed in the previous subsection, though here we mean knowledge of non-moral facts, as opposed to understanding of moral concepts. It also has some relation to memory, insofar as part of having knowledge of some subject involves the ability to bring to mind previously-entertained beliefs. Still, it goes beyond mere memory by requiring further conditions of understanding that allow people to properly appreciate and deploy the relevant facts.^6^

### Openness to Revision

Recognizing faults in one’s reasoning processes is not very useful if one does nothing about it. For this reason, Rawls required competent judges to be open to revision. This can come in at a number of levels – accepting the surprising implications of one’s views, attending to reasons for and against those views, and most importantly being willing to change one’s views after careful reflection.^7^

Openness is a motivational feature that contributes to moral reliability. But it is not the sort of motivational issue that operates at the point of action, as in akratic cases where one acts in a way one believes one should not. Rather, it is a more theoretical motivation to revise one’s moral ideas in the face of compelling reason to do so. To be sure, it is hard to give up one one’s ideas and commitments. One becomes attached to them, personally invested in their truth and value. And, perhaps, some conservatism can be justified – constantly changing one’s ideas can lead to interpersonal unreliability and a fragmented sense of self. But without openness to revision in the face of what one takes to be devastating flaws in one’s judgments, any attempts to revise and improve one’s pre-theoretical views through reasoning would be doomed to failure. Moral progress becomes impossible.

This importance of openness to revision for moral reliability can be easily illustrated. Suppose someone identifies an inconsistency between two moral intuitions. Previously, I have been assuming that something has to give – one will be abandoned, the other retained. But someone could instead simply choose to live in logical contradiction. There is nothing physically stopping them from doing so (in contrast with logical contradictions of actions), and perhaps pride or personal attachment to one’s own ideas makes the option of living in contraction appealing. This decision, though, comes with severe costs: it shuts off a golden opportunity for the person to develop a more reliable view. Insofar as someone cares about being moral, they should be willing to make changes in such circumstances.^8^

Generally, any cases where a person refused to change (adding a new moral judgment or altering/abandoning a previously held one) after undergoing a good reasoning process would undermine their moral reliability. If someone flatly refused to ever change their judgments, any moral improvement of those judgments would be impossible. What could possibly justify such intransigence? Perhaps if the person had reached the pinnacle of human moral thought, there would be no need for further change because improvement is impossible. But reaching such a pinnacle is not plausible, and it is much less so if someone in such a position nevertheless faces a critique of their views that they recognize as devastating.^9^

### Empathetic Understanding

The final criterion laid out by Rawls is what he calls sympathetic knowledge. The main characteristic of this capacity is a sort of psychological competence, the ability to understand and appreciate the interests at stake in various circumstances. But the terminology of sympathy is somewhat misleading. Wispé [14] notes an important psychological distinction between sympathy and empathy. Sympathy typically implies an awareness of someone’s suffering combined with a desire to alleviate it. Empathy is a deeper state of appreciation of someone’s subjective experiences. Because promoting sympathy would presuppose a particular normative framework (we should alleviate others’ suffering), it is unsuitable for our procedural model. Empathy is more neutral, and more clearly identifies a feature of reasoning – the understanding of others’ situation. To be precise, then, we will focus on empathetic understanding.

The easiest way to obtain empathetic understanding is for the judge to actually undergo the experience in question. If one is inquiring into the morality of torture, for instance, having undergone torture oneself would give special (if not decisive) insight into the harms at issue. But direct experience is perhaps too high a bar; demanding discussants of torture undergo torture themselves would be unreasonable as well as impractical. Rawls acknowledge this and notes that “no man can know all interests directly.” (ibid, p. 179)^10^ He suggests a more reasonable standard when direct experience is absent would be “imaginative appraisal.” (ibid) What this consists in is not developed, but it is indicative of a plausible capacity contributing to moral reliability. This is, more or less, the ability to seriously put oneself in another’s shoes; to consider and internalize (to the extent one is able) what they are undergoing, in various circumstances. The imagination may not constitute actual experience, but it should lead to a reasonable amount of understanding of said experience.

Empathetic understanding, like empirical competence, will play an important role in moral deliberation by improving the reliability of non-moral claims integral to moral thought. Returning to the example of torture, it will be a great asset for a moral reasoner to be able to understand and appreciate (even if only to a limited degree) the experience of the tortured. If torture is to be weighed up against other social goods like preventing terrorism, people who can empathetically appreciate just how bad torture is will be able to produce more reliable weightings. And if one is arguing for absolute prohibition, a better understanding of the nature of torture through empathy will be integral to capturing what (if anything) makes the nature of the act so wrong as to be barred without exception. This is indicative of empathy’s broader role in moral reasoning, where internal dynamics are often as crucial as external factors.

One could go further, as Rawls does, and emphasize that empathetic understanding should be of a certain sort: judges should treat and react to the psychological states of others as they would their own. This is too strong a criterion. One’s own reaction to certain psychological states are not necessarily indicative to others’. For example, some people might have a higher threshold for pain (or be masochists), such that the same experiences are to be interpreted differently; or they might have different appreciation of language like racial slurs; or they may have different value sets that demand emphasis or de-emphasis of certain states like homosexual attraction;^11^ and so on. We should not presuppose that the judge’s reaction to an experience is indicative of how all would or should respond. Still, there is a reasonable motive for wanting to employ this methodology – to avoid selfish privileging of one’s own interests over those of others. This implies a further distinct criterion to which we will now turn, concerning bias.

### Bias Avoidance

Though it is not delineated as a separate category, at several points Rawls suggests that judges should seek to avoid various sorts of biases. He places most emphasis on self-interest bias, where a moral judgment is coloured by whatever would make oneself better off. Such self-interest bias is problematic in itself, to be sure, but it is also indicative of a broader class of biases that can impede moral reasoning. Avoiding biases would generally lead to an improvement in moral reliability.^12^ For present purposes, we will use the following definition of bias: taking factors into account in a moral judgment that are not relevant to that moral judgment.^13^ This captures the essence of what goes wrong in biases – racists are taking race into account when they should not. Straightforwardly, judgments with greater reliance on relevant inputs will be more reliable – this follows from the nature of relevance, an identification of the factors that do indeed bear on the veracity of a given claim. This is more or less in line with Aristotle’s Equality Principle, where one must treat like cases alike unless there is a morally relevant difference.

It might be questioned whether this account is truly procedural, though: it presupposes a certain notion of what factors are morally relevant to certain judgments, and what are not. That is a substantive issue, and might also be seen as overly question-begging.

In response, we would first say that the substantive assumptions in the definition of bias are not necessarily problematic. The reason we seek a procedural account of moral reliability is to avoid presupposing the rightness of certain outputs and getting bogged down in controversial assumptions. But presupposing the moral relevance of certain factors does not presuppose the correctness of certain outputs. The substantive assumptions in play are second-order, after all. Reducing or eliminating the influence of certain factors still leaves wide open the actual judgments one makes. What’s more, it can be expected that many forms of bias (like racism) will be uncontroversially problematic; it will not impede practical implementation to make certain second-order assumptions.

Still, some sources of bias will be controversial – for instance, it is debatable whether giving priority to one’s co-nationals is a bias or fair priority. An alternative solution, then, is to narrow the definition even further: biases occur when one takes factors into account that are not morally relevant, *by one*’*s own lights*. By relying not on objective standards but one’s own, it maintains procedural neutrality. However, this account is rather permissive. It prevents us from criticizing as biased people who really consider some factor relevant. For instance, the thoroughgoing racist who has an internal view that whites just are morally superior to other races would not be biased in taking race into account in various moral judgments. For this reason, the account may not serve as an adequate analysis of the notion of bias as it is typically deployed. Yet, in practice this would not generate overly-permissive results very often. Such thoroughgoing racists are relatively rare in modern society; actual racism much more manifests itself as people unintentionally taking race into account even when they accept that they should not.^14^ Other sorts of biases are similarly uncontroversial in their standards: how you frame a question should not matter to one’s opinion of it; one should not hold oneself to different moral standards as that of others; one should not privilege one’s relations over others in the public sphere; and so on. Given general acceptance of such standards, attribution of bias will be acceptable in such cases. Controversial cases like nationalism will admittedly only be partly accounted for, but a wide array of uncontroversial forms of bias will remain as targets.

We believe that substantive second-order standards for moral reliability will not interfere with the procedural nature of the present account. If one disagrees, we would suggest adopting the alternative neutral account instead. Bias avoidance will be more a narrower category and the extent to which improvement in this domain improves moral reliability would be more limited. But given that many biases are not even internally endorsed, the actual effect of such narrowing may well be relatively minor.

With this definition in mind, the link between avoiding bias and moral reliability should be clear. By removing the pernicious influence of irrelevant factors in moral reasoning, the proportion of remaining factors that are indeed relevant will increase. As relevant factors are more likely to lead to good judgments than irrelevant factors, reliability should in turn be improved.

And what form should bias avoidance take? This is a more empirical question, but some suggestions can be made here. Promoting bias avoidance can in part consist in helping people recognize such conflicts (such as by making their standards more personally salient or explicitly pointing out such standard-violations when they occur), as well as techniques that might reliably reduce instances of erroneously taking various factors into account. So, for one who takes racism to be problematic, a program of sensitization to other races may count as an indirect moral enhancement insofar as it helps people conform their specific judgments to their standards over when race can be taken into account.

### Taking Stock

The aforementioned six features - logical competence, conceptual understanding, empirical competence, openness, empathetic understanding and bias avoidance – all contribute to procedural moral reliability. They could do so each on their own, but there is a certain synergy between them – for example, empirical competence informing the premises of logical arguments, which leads to a change in thought thanks to the agent’s openness. They share a common rationalist thread, and might appear to be Platonic in structure: moral judgments are subject to and significantly controlled by considered moral reasoning. But the rationalism of this approach is not meant to be exclusive. We are open to the notion that other features might contribute independently to moral reliability. These features are readily identifiable, however, and fit nicely into a proceduralist paradigm.

A further advantage of this approach is that, due to its relatively minimal commitments, it is compatible with a wide array of normative and metaethical views. There is not space to fully develop this here, but there is some overlap with Aristotle’s *Nicomachean Ethics* [17] and Hume’s contentions in *On The Standard of Taste* [18] .^15^ One also need not adopt an overall Rawlsian framework to accept the merits of our approach; though Raz [19] rejects Rawls’ reflective equilibrium, even he accepts the importance of logical constraints and bias avoidance for moral reasoning. In this way, it is a *minimally* procedural account – taking on board a relatively small set of features that most plausible accounts of moral reasoning should be able to accommodate. This broad acceptability should aid in practical uptake and ability to actually improve moral reasoning.^16^

## Garbage In, Garbage Out?

Before moving on to the implications of our view, we will address an important objection to the above account. The neutrality of our procedural approach has until now been considered an asset, but it has a crucial flaw as well. Procedural improvement in moral reasoning is worthless if the aspects of moral reasoning not subject to procedural constraints (such as moral inputs) are deeply misguided. If we remain neutral on the soundness of those inputs, the procedural improvements will do little to improve the reliability of people with flawed inputs (be they intuitions, perceptions, or something else). In fact, improved procedures on somebody with flawed inputs might make their moral judgments less reliable.

Consider the case of Huckleberry Finn. In Twain’s novel of the same name, Huck is deliberating over whether or not to return his friend Jim, who is a runaway slave in the antebellum South, to Jim’s master. According to one reading [20], Huck thinks that the weight of reasons are on the side of turning Jim in – however, the non-reasons-responsive sympathy for his friend prevents Huck from going through with it. Huck ends up doing the right thing and allows Jim to go free. But, the worry goes, what if Huck was a better reasoner, and more open to revising his judgments on the basis of the weight of reasons? He may well have recognized the force of what he took to be good reasons, excluded the ‘bias’ of friendship from consideration, and turned Jim in.

There are two related worries associated with this sort of example. One, we might worry that moral premises not subject to procedural critique (including an endorsement of slavery) are flawed, with better understanding of the implications of one’s views leading to even more misguided conclusions. Two, when faced with an evident conflict in judgments, we have no guarantee that people will make the right choice. While arguably Huck chose correctly, that is based on substantive assumptions a procedural account is meant to exclude. Is there any procedural reason to think that people will reliably choose correctly?

We can say two things in response. Firstly, it is true that strict logical coherence may do badly in Huckleberry Finn-type cases. However, there are procedural reasons to hope for improvement in such cases on the other domains listed. Moral endorsement of slavery may be based on faulty empirical assumptions – racist claims about inherent superiority or natural fittingness that, with greater understanding, can be rejected. Relevant reasons may not, when properly thought through, favour turning Jim in. Openness could lead Huck to be more likely to reject his sympathy, but also the social morality which he seems to be ambivalent towards anyway. Empathetic understanding would allow greater appreciation of Jim’s plight, ensuring his interests and the suffering he would have to undergo as a slave are fully taken into account. And, of course, bias avoidance could help rid Huck of any racist predilections that undergird the morality that justifies the enslavement of blacks in the first place.

Secondly, cases like Huckleberry Finn only pose a serious threat to our account of moral reliability if one has a particularly pessimistic view of moral decision-making. On a merely neutral view (agents are as likely to correct in the right direction as the wrong one), there is still the elimination of a clearly incorrect set of opinions, namely, the jointly-held incorrect claims. The agent’s (moral) epistemic position will be expected to improve in at least one regard (fewer sets of opinions that are definitely wrong), even if we could not be confident that the new set are correct. They are, in that sense, more reliable.

If one thinks agents make systematic errors, the same does not apply. Systematic mistakes in correction of incoherent views would lead to an even worse epistemic state. On such a view, coherence might well be a positive evil, one that seriously threatens moral reliability. However, this view is very difficult to justify. Why would people be systematically choosing incorrectly? One might point to particular systematic biases, but on our account bias avoidance is already part of the picture of improved moral reliability. One might be an error theorist, but there’s no particular reason people will systematically choose positive moral claims (which the error theorist would claim are generally incorrect) over negative moral claims (which they may accept). In the absence of a plausible a sound basis of the pessimism of the view, the objection need not be taken seriously.

It may nevertheless be objected that the preponderance of errors in moral judgments (and subsequent injustices) do not occur at the procedural level, but due to flawed normative inputs. Our approach, while affecting some gains at the margins, would on this view not have a substantial impact on moral reliability. This worry, though, is difficult to evaluate. How can we tell the proportion of the sources of errors? We could try to identify the sources disagreements, to see whether they are really procedural or deeply value-laden. But even deep values will be subject to procedural critique (they may be incoherent, ill-informed, closed-minded, etc.).

More positively, it is indeed plausible that many moral disputes occur at the procedural level. Disagreements over the extent to which racism and sexism are a problem in our society are not primarily over whether racism and sexism are bad, but rather the extent to which such biases exist and how harmful they are – addressable by the features of empirical competence and empathetic understanding. Hypocrisy, a form of logical inconsistency, is a common and acceptable critique in normative discourse. And political discourse itself, insofar as it serves some transformative purpose, relies at least to some extent on the willingness of people to be open to changing their views; converse closed-mindedness is arguably a problem for real political progress. Procedural issues are not the whole story, but they have a major role to play in various arenas.

## Conclusions

Moral reliability is an important notion that can offer an amenable way forward for critics of other forms of moral enhancement. We have offered a procedural framework that identifies six features contributing to moral reliability: logical competence, conceptual understanding, empirical competence, openness, empathetic understanding and bias avoidance. This account succeeds on four dimensions: it avoids controversial normative assumptions; the features can be expected to contribute to moral reliability; it focuses on agents themselves; and the account can provide useful guidance in a variety of contexts.

As the present paper is aimed at developing a framework for procedural moral enhancement, we have not been able to delve very deeply into those practical implications. Several should be clear enough, though. Our framework suggests a useful form of procedural moral education, one that can both lead to better moral deliberators while not imposing particular values on students.^17^ Already, certain capacities we identified – empirical competence in particular – are a standard part of modern curricula. Others, though, are lacking. Greater emphasis on logic classes, bias awareness/avoidance and even empathy training in schools may be warranted.

In addition, a variety of roles require good moral deliberators – ethics committee members, judges and jurors, even politicians. There are already procedures in place to identify competencies for those various positions, but our framework suggests reasonable further criteria for the selection - supplementing existing criteria, not replacing them. Like in education, some of these procedures already match parts of our framework; jury selection often involves questions trying to tease out potential biases. But perhaps we should look at other sorts of tests – improved juror empathy, for instance, would allow for better appreciation of the sincerity of witness statements, while logical and conceptual competence may assist in following lawyers’ sometimes complicated arguments.

More prospectively, our proposal suggests a promising approach to moral bioenhancement. Beyond attempts to improve motives or behaviours, we should look at improving deliberative processes themselves. This allows one to avoid the controversial issue of imposing one’s values on individuals while still promising moral improvement. Many of the capacities we identify should be susceptible to biological improvement, at least in principle – but much more research needs to be done in this area before interventions can be seen as viable.^18^

It would not be unfair to observe that our procedural account is philosophical in nature. In fact, one could characterize procedural moral reliability as designed to make people better philosophers – features like logic and conceptual analysis, after all, are hallmarks of the analytic tradition. This is no coincidence. As mentioned above, we do not think that philosophers are, in virtue of their training, better people than the rest of the population. However, we do have some confidence that philosophical approaches to moral problems are at least somewhat reliable at coming to correct moral judgments – at least, they are more reliable than unreflective alternatives. And that more minimal claim is all we propose in the present paper. Procedural moral reliability does not identify moral paragons or the unquestionably correct theory. It simply identifies features that lead individuals to be more morally reliable, all else being equal, than they otherwise would be.

## Footnotes

1 Our argument bears some similarities to Jefferson et al. [2], “Enhancement and Civic Virtue,” especially its contention that cognitive enhancements can have benefits for moral deliberation. However, that argument focused on desirable political side-effects of more general biomedical enhancement rather than a general framework for moral enhancement (as we offer). Moreover, even if the argument were reformulated as a form of general moral enhancement, its reliance on a thick concept of civic virtue makes it substantive in nature – in contrast to our own procedural approach.

2 One could try to avoid these issues by focusing enhancement on a few uncontroversial norms, like wanton aggression or selfishness. Even if that strategy succeeds, it would necessarily be limited. Our procedural framework has the advantage of leading to expected moral improvement in areas that are controversial. Arguably, those controversial areas are the ones where we could hope for the most amount of progress and improvement. This is not to rule out the viability of more substantive approaches, or a combination of substantive and procedural interventions. We are arguing, more moderately, that procedural approaches can make significant combinations to the reliability of people’s moral judgments.

3 That is not to say there is no correlation between general intelligence, or even computational competence, and moral behaviour; some correlation, for instance, has been detected between altruism and intelligence ([11, 12]. However, such studies do not isolate specific aspects of intelligence, are merely correlational and not causal, do not address procedural reliability per se and focus on only one narrow aspect of morality (altruism). In any case, for the purposes of this paper we will focus on the theoretical basis for a link between moral reliability and reasoning capacities.

4 Dialethists, who accept the possibility of true contradictions, might question the emphasis on coherence. And whatever the merits of dialethism, this might undermine the procedural nature of the approach – we presuppose the negation of dialethism, a substantive position. We have two responses. One, the rejection dialethism is not a output of moral reasoning, so is strictly outside the scope of the sort of judgment we do not want to presuppose. Two, dialethists hardly reject the importance of logical competence anyway; they simply have an alternative view of it. Dialethists should also embrace better logical competence, at least in theory, as that will allow people to better understand how to engage with true contradictions.

5 This assumes that people will be motivated to correct judgments when faced with inconsistencies. In practice, some may be content to let such inconsistencies stand – not because they are dialethists, but simply because they do not care enough to change their views. Some of this will be addressed in the section on openness to revision below, but this points to broader cases of akrasia – acting in a way one accepts one should not. While akrasia reduction was not included in our present framework, because it does not easily fit with our focus on judgments, we accept that it may be a good supplementary means of achieving procedural moral improvement.

6 Other potential capacities in this category include imagination, which John Stuart Mill emphasizes as crucial to discourse in *On Liberty*, as well as the ability to discern expertise, and ability to work with sources of knowledge like libraries and internet encyclopedias.

7 There is some connection between failure to be open to revision and the social intuitionist model of moral judgment promoted by Jonathan Haidt [13]. According to Haidt, our moral judgments originate from intuitionist, emotional reactions which are then rationalized, post-hoc. Moral intransigence arises because explicit reasoning and argumentation operates at the level of rationalization, rather than emotional processing. But things are not completely hopeless – Haidt (like Hume) accepts that reasoning processes (especially in a social setting) can indeed have some causal influence on judgments, even if they are secondary to intuitive reactions. Our own approach focuses on the influence of those reasoning processes, which we are presently arguing can lead to reliable improvements.

8 Openness might also include, not only willingness to change, but also willingness to learn, to seek out and engage dialectically with opposing viewpoints that would bolster one’s reasoning process.

9 One might think this is in tension with certain dogmatic religious traditions that resist change and revision. However, such religions are often very encouraging of openness. Specifically, then rely on openness to convert non-believers to their faith, and continued openness is needed to grow in that faith. They may frown on openness to rejecting the faith, but they would not do so thoughtlessly or in wilful ignorance of decisive refutations; rather, they rely on considered judgments that their faith is indeed sound and refutations are unsuccessful.

10 Interestingly, Mill developed the former sort of strict familiarity as a criterion of a competent judge in *Utilitarianism* – without expanding this, as Rawls does, to empathetic capacities more generally. But given Mill’s insistence on the importance of imaginative capacities elsewhere in his work, the present case for empathetic knowledge may be acceptable on Mill’s framework as well.

11 To make this more vivid, a man who views homosexuality as immoral may well think discrimination against himself, were he homosexual, would be perfectly justified and appropriate, with any resulting harms perfectly deserved. But this would obscure an important insight empathy could bring to the man, namely the harm that *others* experience from such discrimination.

12 See Sinnott-Armstrong [15] for a similar point. Sinott-Armstrong is careful to note that mitigation or removal of bias is not sufficient to warrant overall trust in the agent’s judgment, and we agree. However, this can be very indicative on their own of the relative reliability of reasoners. We have good reason, all else being equal, to place higher confidence in the judgments of A over B when A is less susceptible to bias than B.

13 This bears some similarity to Nozick’s understanding of bias as the uneven application of standards ([16], p. 103), but is narrowed somewhat to avoid including all cases of inconsistency.

14 This approach still has some resources to address thoroughgoing racists. Better appreciation of empirical facts concerning races as well as more thorough understanding of the concept of race itself along with moral notions like fairness and equality could all be leveraged in an effort to morally improve the thoroughgoing racist.

15 Despite its rationalist structure, our account is also compatible with a sentimentalist approach; even sentimentalists like Hume accept the use of rationality and reasoning in making moral judgments.

16 It is a further question whether this framework runs afoul of feminist critiques of rationalist epistemology, e.g., that emphasis on rationality reinforces a specifically masculine way of thinking. Again, we cannot fully develop a response here; we would just briefly suggest that our identified features are not exclusively rationalist. While one of our features is logical competence (very rationalistic), another involves empathy (more sentimentalist). Other features like openness and bias avoidance should also be amenable to feminist paradigms.

17 In this way, we can maintain liberal neutrality in our moral education paradigm, something that some modern Rawlsians emphasize (e.g., Waldren [21] and Ebels-Duggan [22]).

18 Our suggestions vis-à-vis bioenhancement, then, should be seen as compatible with Persson and Savulescu [23] and [24]): we should engage in further research into interventions that could improve the identified capacities. The arguments of this article are neutral, though, on whether we should also pursue research into non-procedural moral bioenhancement.
